# From student organizer to leader: the role of event planning in management skill development

**DOI:** 10.31744/einstein_journal/2025EDS3

**Published:** 2025-09-29

**Authors:** Sophia Luiz Calegaretti, Emily Braun, Claudio Luiz Lottenberg

**Affiliations:** 1 Hospital Israelita Albert Einstein Faculdade Israelita de Ciências da Saúde Albert Einstein São Paulo SP Brazil Faculdade Israelita de Ciências da Saúde Albert Einstein, Hospital Israelita Albert Einstein, São Paulo, SP, Brazil.; 2 Hospital Israelita Albert Einstein São Paulo SP Brazil Hospital Israelita Albert Einstein, São Paulo, SP, Brazil.

In common parlance, the term "event" often evokes images of celebrations, ceremonies, or large gatherings meant primarily for leisure. However, when considered in the academic environment, event planning can be understood as a pedagogical tool that fosters organizational, interpersonal, and leadership skills among students. Far from being limited to logistical tasks such as scheduling rooms, event planning becomes a formative process in which students learn to manage resources, coordinate teams, and handle unforeseen challenges. In this sense, organizing an academic or institutional event represents much more than an extracurricular activity: it is an opportunity to cultivate essential management skills that can shape future leaders.

The organization of events can be understood as a training ground for what Pierre Bourdieu^( 1 )^ describes as a "managerial habitus." Just as the scientific habitus is gradually internalized through research practice, the managerial habitus is formed when students repeatedly engage in planning, negotiation, and leadership within real social contexts. This set of implicit dispositions begins to guide behavior and decisions in leadership situations – often unconsciously – while equipping students to act with greater confidence in professional environments.

This dynamic aligns with Alexander Astin's Theory of Involvement,^( 2 )^ which highlights that student development depends not only on the promotion of extracurricular activities by institutions, but also on the degree of active participation of students themselves. The investment of time, cognitive effort, emotional energy, and physical presence fosters deeper learning, more solid skill acquisition, and significant personal growth. By engaging in such activities, students shift from a passive stance toward knowledge to becoming active agents of their own learning, building stronger connections with academic life, peers, professors, and professional networks.

The skills developed through this active involvement are diverse. They include the capacity to manage organizational demands – such as planning activities, setting priorities, and balancing time – as well as the ability to communicate and collaborate effectively, in which empathy and flexibility are central to handling interpersonal dynamics and resolving conflicts. Students also cultivate resilience and adaptability, essential for coping with pressure, overcoming setbacks, and maintaining motivation in challenging circumstances. Consequently, they conclude their academic journey with greater responsibility, self-confidence, and an internalization of values such as commitment – attributes that enhance their preparation for professional life.

Max Weber's distinction between the ethics of conviction (Gesinnungsethik) and the ethics of responsibility (Verantwortungsethik)^( 3 )^ further illuminates the ethical dimension of student leadership. While conviction motivates students to act in accordance with ideals of collaboration and academic commitment, responsibility demands reflection on the concrete consequences of their decisions for peers, institutions, and communities. The interplay between these ethical orientations strengthens the formative character of event planning, requiring critical reflection and prudent judgment in the exercise of leadership.

Thus, the transition "from student organizer to leader" is not merely metaphorical, but a tangible process through which young professionals internalize values of responsibility, resilience, and collaboration. Event planning enables them to envision themselves not only as students, but as active agents capable of leading teams, managing complexity, and promoting transformation. In doing so, they contribute both to their personal development and to the consolidation of stronger academic communities and professional networks.
